# Hyperacusis in Children with Attention Deficit Hyperactivity Disorder: A Preliminary Study

**DOI:** 10.3390/ijerph17093045

**Published:** 2020-04-27

**Authors:** Massimo Ralli, Maria Romani, Alessio Zodda, Francesca Yoshie Russo, Giancarlo Altissimi, Maria Patrizia Orlando, Maria Gloria Cammeresi, Roberta Penge, Rosaria Turchetta

**Affiliations:** 1 Department of Sense Organs, Sapienza University of Rome, Viale del Policlinico 155, 00100 Rome, Italy; alessio.zodda@libero.it (A.Z.); francescayoshie.russo@uniroma1.it (F.Y.R.); giancarlo.altissimi@uniroma1.it (G.A.); mariapatrizia.orlando@uniroma1.it (M.P.O.); mariagloria.cammeresi@uniroma1.it (M.G.C.); rosaria.turchetta@uniroma1.it (R.T.); 2 Center for Hearing and Deafness, University at Buffalo, Buffalo, NY 14214, USA; 3 Department of Pediatrics and Child Neuropsychiatry, Sapienza University of Rome, Viale del Policlinico 155, 00100 Rome, Italy; maria.romani@uniroma1.it (M.R.); roberta.penge@uniroma1.it (R.P.)

**Keywords:** hyperacusis, attention deficit hyperactivity disorder, autism spectrum disorders, hearing

## Abstract

The association between hyperacusis and developmental disorders such as autism spectrum disorders has been extensively reported in the literature; however, the specific prevalence of hyperacusis in attention deficit hyperactivity disorder (ADHD) has never been investigated. In this preliminary study, we evaluated the presence of hyperacusis in a small sample of children affected by ADHD compared to a control group of healthy children. Thirty normal hearing children with a diagnosis of ADHD and 30 children matched for sex and age were enrolled in the study. All children underwent audiological and multidisciplinary neuropsychiatric evaluation. Hearing was assessed using pure tone audiometry and immittance test; ADHD was diagnosed following the Diagnostic and Statistical Manual of Mental Disorder criteria. Hyperacusis was assessed through the administration of a questionnaire to parents and an interview with children. Hyperacusis was diagnosed in 11 children (36.7%) in the study group and in four children (13.3%) in the control group; this difference was statistically significant (*p* = 0.03). The preliminary results of this study suggest a higher presence of hyperacusis in children with attention deficit hyperactivity disorder compared to control children. More studies on larger samples are necessary to confirm these results.

## 1. Introduction

Hypersensitivity to sound (hyperacusis) is described as a reduced tolerance to sounds of average intensity, sometimes accompanied by painful sensitivity to ordinary environmental sounds, with perceptual, psychological and social dimensions [1]. The causes of hyperacusis are still debated; recent imaging studies indicate the hyperexcitability of specific brain areas as a common finding in these patients [2,3,4,5,6,7,8]. Available evidence suggests that hyperacusis could originate from functional alterations in the central nervous system correlated to generalized hyperactivity following an increase in the gain of auditory and extra-auditory pathways and to greater anxiety or emotional response to sound [5,7,9,10]. There is a growing awareness that, although more prevalent during adulthood, children may also experience hyperacusis [11,12,13,14,15]. Data on the prevalence and characteristics of this condition during childhood are fragmented; the estimated prevalence varies between 3.2% and 17.1% of the pediatric population [13,16]. Hyperacusis can have significant effects on children’s behavior; sounds can be perceived as painful and trigger avoidance conducts, leading to isolation and influencing social interactions and daily activities with potential consequences on education, communication and learning [17,18].

Attention deficit hyperactivity disorder (ADHD) is a childhood-onset neurodevelopmental condition characterized by marked, persistent and maladaptive age-inappropriate levels of inattention, hyperactivity, and impulsivity [19,20]. The estimated incidence of ADHD is 7% of the pediatric population and 4% of the adult population; the rates of the disorder in the general population have been increasing over the past decade also thanks to more accurate diagnostic protocols [21,22,23]. ADHD is mainly diagnosed during primary school; symptoms include common traits with autism spectrum disorders (ASD)—although ADHD is not part of the autism spectrum—and include accentuated restlessness and evident presence of cognitive symptoms, such as inattention, easy distractibility, impulsiveness, scholastic difficulties, avoidance of cognitive tasks, impulsive reactions, low self-esteem and sometimes low olfactory threshold [24,25,26,27]. ADHD is the most common comorbid psychiatric condition in patients with ASD, with comorbidity rates as high as 70% [28]. Similarly, ASD traits can be found in children with ADHD, with nearly 12% having a diagnosis of ASD [29]. The causes underlying this relationship are still unclear, although specific genetic variants have been suspected [30].

The association between hyperacusis and developmental disorders such as ASD has been extensively reported in the literature; a higher prevalence of hyperacusis has been reported in ASD and Williams syndrome [17,31,32,33,34,35,36]. However, the prevalence of hyperacusis in children with ADHD has never been specifically investigated. The aim of this preliminary study is to evaluate the prevalence of hyperacusis in a cohort of normal hearing pediatric patients affected by ADHD compared to a control group of healthy children matched for age and sex.

## 2. Materials and Methods

### 2.1. Study Sample

The study participants consisted of 60 children of both sexes, aged between 4 and 12 years, with clinically normal hearing. Children were divided into two groups: a study group of children with neuropsychiatric diagnosis of ADHD combined type (*n* = 30), and a control group of healthy children matched for age and sex (*n* = 30).

Clinically normal hearing was defined according to the American Academy of Otolaryngology and American Council of Otolaryngology [37] as an individual hearing threshold ≥25 dB HL at frequencies from 250 to 4000 Hz at the octave scale in both ears.

ADHD was diagnosed following the fifth edition of the Diagnostic and Statistical Manual of Mental Disorder (DSM-5) criteria, and only children with a diagnosis of ADHD, combined presentation, were included [38].

Exclusion criteria were the presence of hearing loss, recent episodes of otitis media with effusion, previous ear surgery, ADHD with predominantly inattentive or predominantly hyperactive/impulsive presentation, a Full-Scale Intelligence Quotient <85, and concomitant neuropsychiatric comorbidities.

The study was approved by the local Ethic Committee of our University Department (14/2019, 23.07.2019) and was performed in accordance with the Helsinki declaration and its amendments. Informed consent was obtained from the parents of the participants.

### 2.2. Diagnosis of Attention Deficit Hyperactivity Disorder

Diagnosis of ADHD was performed in the Department of Human Neuroscience of our university. Children were referred on the recommendation of parents, teachers or pediatricians. All children and parents underwent an initial interview with a child neuropsychiatrist who collected the child’s history, followed by an evaluation performed by a multidisciplinary team of experts comprising a neuropsychiatrist and two psychologists. The diagnosis of ADHD was made following the DSM-5 criteria for ADHD, combined presentation. Treatment was individually designed and adapted to the child’s age and condition and may have included psychostimulants and behavior therapy, counseling, social skills training, psychotherapy and child and family education services.

### 2.3. Auditory Evaluation

Auditory evaluation was performed in the Department of Sense Organs, Pediatric Audiology Unit of our university. All patients underwent full otolaryngology examination, pure tone audiometry (PTA), and acoustic immittance test. PTA (Piano Clinical Audiometer Inventis, Padua, Italy) was measured at frequencies of 125, 250, 500, 1000, 2000, 3000, 4000, and 8000 Hz. Acoustic immittance measures were performed to measure the functional integrity of the eardrum and middle ear anatomy using tympanometry tests and acoustic stapedial reflex tests (AT 235 Tympanometer, Interacoustics, Middelfart, Denmark). 

### 2.4. Diagnosis of Hyperacusis

Hyperacusis in children was evaluated through the administration of a questionnaire to parents (PQ) (Table 1) and an interview with children (CI) (Table 2). Both questionnaire and interview were modified from Coelho et al. (2007) and translated into Italian, as already done in a previously published study from our group [11].

The PQ was divided into two sections: the first examined hypersensitivity to everyday sounds reported by parents using four short and concise multiple-choice questions investigating the relationship that their children has with sounds. Each question had three possible answers: YES (4 points), DON’T KNOW (2 points) and NO (0 points) (Q1–4). The second section included six questions investigating the most common reactions of their children to sounds (Q5–10). A child was considered hypersensitive to sound if he obtained a score >8 points for questions 1–4. 

The CI included three main questions investigating hearing loss, the presence of tinnitus and subjective hypersensitivity to sounds. Furthermore, it was asked of the children to indicate which sounds annoyed them from a list of 20 sounds. If a positive answer was given to the question “Are you bothered by any kind of sound or noise?” and the child indicated five or more sounds from the list of 20 sounds as annoying, the child was classified as “hypersensitive to sound.”

**Figure 1:**
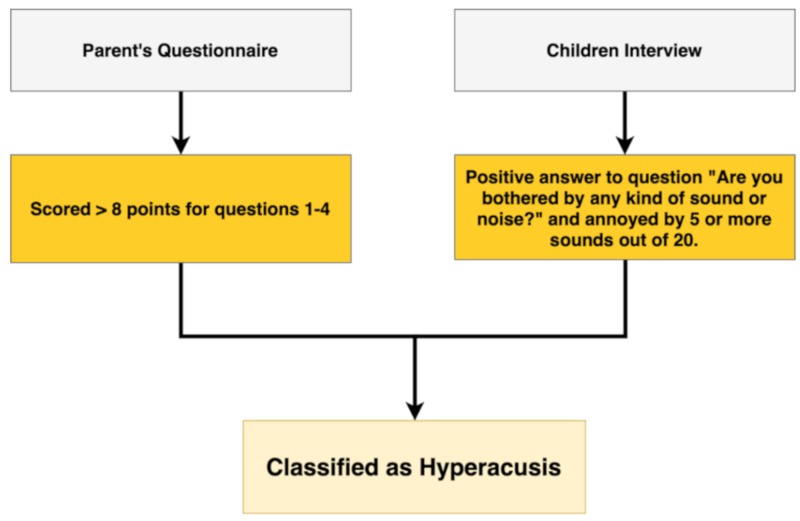
Flowchart showing the criteria used to classify questionnaire and interview data and to define hyperacusis. Children were considered hyperacusic if they scored hypersensitive to sound at both the parent’s questionnaire and children’s interview.

Children were considered hyperacusic if they scored hypersensitive to sounds in both the PQ and the CI. The flowchart (Figure 1) outlines the criteria used to classify questionnaire and interview data and to define hyperacusis.

### 2.5. Statistical Analysis

Descriptive analysis was used to define the main clinical and demographic characteristics of the patients. Data were expressed as means ± standard deviations. The statistical analysis was conducted using the Unpaired *t* test to highlight differences in basic characteristics (age, sex) between groups (not expected) and any difference in prevalence of hyperacusis and answers to questionnaires. A *p*-value less than 0.05 was considered the cutoff for statistical significance. Prism Software version 8.3.1 (GraphPad Software LLC, San Diego, CA, USA) was used to perform statistical analysis.

## 3. Results

### 3.1. Patients’ Characteristics 

Thirty children with a clinical diagnosis of ADHD (study group) and 30 children matched for sex and age (control group) were enrolled in the study. In the study group, 25 children were males (83.3%) and five were females (16.7%). Mean age was 7.7 years (range: 5–12 years; SD: 1.78, SE: 0.32). In the control group, 24 were males (80%) and six were females (20%). Mean age was 7.4 years (range: 4–12 years; SD: 1.883, SE: 0.3439). No significant differences were seen for age between the study and control group (*t* = 0.63, *p* = 0.52).

**Figure 2:**
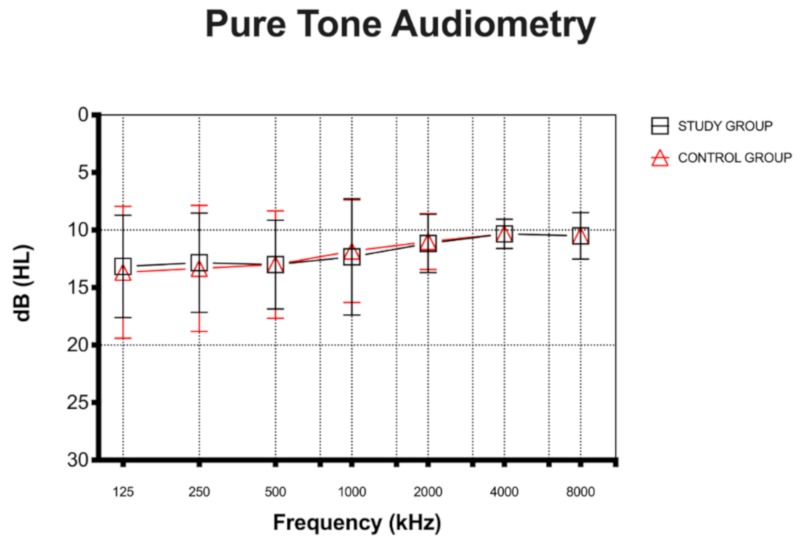
Pure tone audiometry (PTA) in the study and control groups. All children had normal hearing, with an average PTA < 25dB HL for each frequency in the 250–4000 Hz range.

All children in the study and control groups had normal hearing, with an average PTA < 25dB HL for each frequency in the 250–4000 Hz range (Figure 2). 

No significant differences were found for PTA between the two groups (*t* = 0.06, *p* = 0.94). In the study group, 25 children (83.3%) had a type-A tympanogram, three (10%) a unilateral type-C tympanogram and two (6.7%) a bilateral type-C tympanogram. In the control group, 23 children (76.7%) had a type–A tympanogram, three (10%) a unilateral type-C tympanogram and four (13.3%) a bilateral type-C tympanogram.

### 3.2. Parent’s Questionnaire

**Figure 3:**
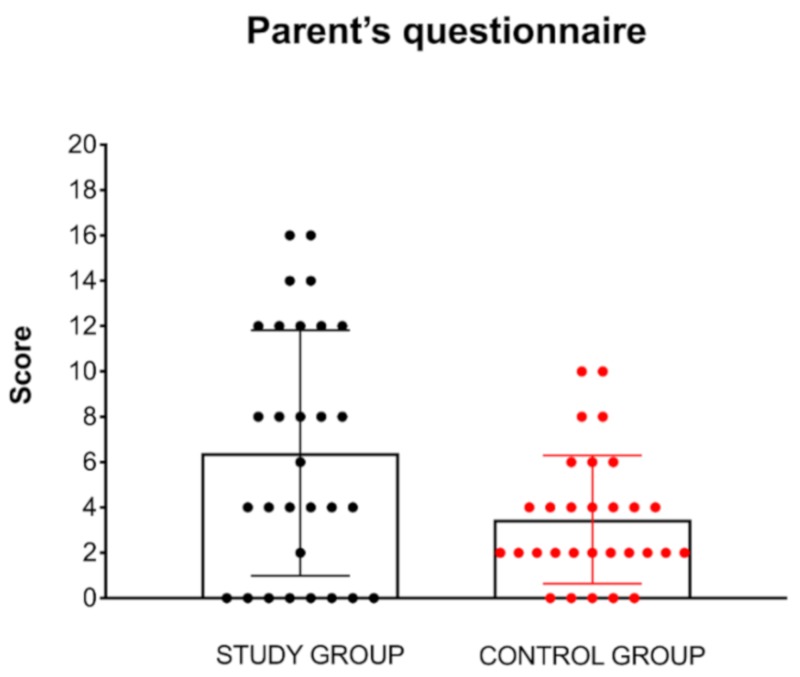
Results of the parent’s questionnaire for hyperacusis. Mean score in the study group was 6.4 compared to 3.4 in the control group. Difference for mean score between the study and control groups was statistically significant (*p* = 0.01).

Mean score for PQ in the study group was 6.4 compared to 3.4 in the control group. Difference for mean PQ score between the study and control groups was statistically significant (*t* = 2.63, *p* = 0.01, *d* = 0.67) (Figure 3).

In the study group, PQ was positive for hypersensitivity to sound (score > 8) in 14 children (46.7%; range 8–16; mean = 11.4) and negative in 16 children (53.3%; range 0–6; mean = 2). In the control group, PQ was positive in four children (13.3%; range 8–16; mean = 9) and negative in 26 children (86.7% range 0–6; mean = 2.7). 

**Figure 4:**
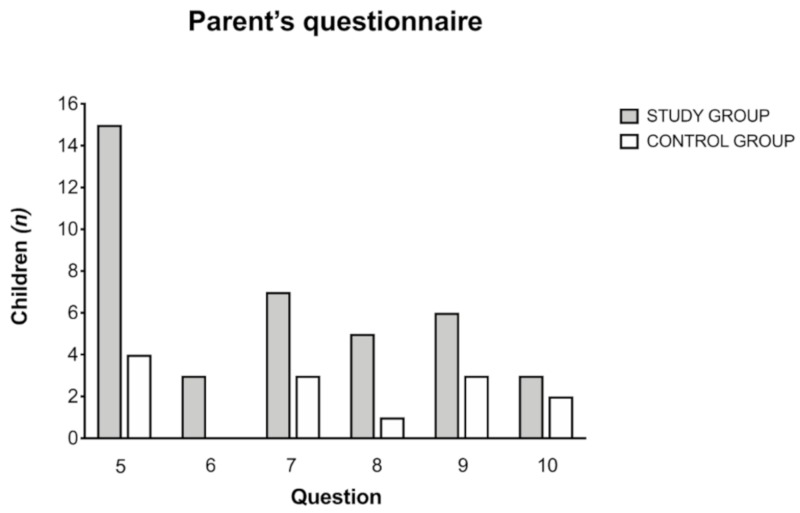
Specific results of questions 5–10 of the parent’s questionnaire for children in the study and control groups. In the study group, a positive response was most found for question 5—cover ears, followed by question 7—escape from sounds, and question 9—saying “it hurts”. In the control group, a positive response was mostly found for question 5, followed by question 7 and question 9.

Specific results of questions 5–10 for both groups are detailed in Figure 4.

In the study group, a positive response was mostly found for question 5—cover ears (50%), followed by question 7—escape from sounds (23.3%), and question 9—saying “it hurts” (20%). In the control group, a positive response was found for question 5—cover ears (13.3), followed by question 7—escape from sounds (10%), and question 9—saying “it hurts” (10%). Difference was statistically significant only for question 5 (*t* = 3.26, *p* = 0.001), while it did not reach significance for question 6 (*t* = 1.79, *p* = 0.07), 7 (*t* = 1.38, *p* = 0.17), 8 (*t* = 1.73, *p* = 0.08), 9 (*t* = 1.07, *p* = 0.28), and 10 (*t* = 0.46, *p* = 0.64).

### 3.3. Children’s Interview

All children in both groups stated that they had normal hearing. Four children in the study group (13.3%) and two in the control group (6.7%) reported experiencing tinnitus (*t* = 0.85, *p* = 0.39), while 25 children (83.3%) in the study group and seven (23.3%) in the control group reported being bothered by any kind of sound or noise (*t* = 5.73, *p* < 0.0001, *d* = 1.47). 

A detailed analysis of the sounds that were reported by children to be annoying (total = 20 sounds) is shown in Table 3. 

In the study group, 14 children were annoyed by 0–4 sounds (46.7%), 11 (36.7%) by 5–9 sounds and five (16.7%) by more than 10 sounds. The most annoying sounds were classroom sounds and screams, followed by bombs and school bells. In the control group, 23 children were annoyed by 0–4 sounds (7.7%), six (20%) by 5–9 sounds and one (3.3%) by more than 10 sounds. The most annoying sounds were screams and thunder, followed by bombs, classroom noise, school bells and firecrackers.

On average, children in the study group were annoyed by 5.4 sounds compared to three in the control group; the difference was statistically significant (*t* = 2.80, *p* = 0.006, *d* = 0.12).

### 3.4. Prevalence of Hyperacusis

Hyperacusis was assessed based on PQ and CI results. In the study group, 11 children (36.7%) scored positive in both the PQ and the CI and were classified as hyperacusic, while in the control group only four children (13.3%) scored positive in both the PQ and the CI (Table 4).

**Figure 5:**
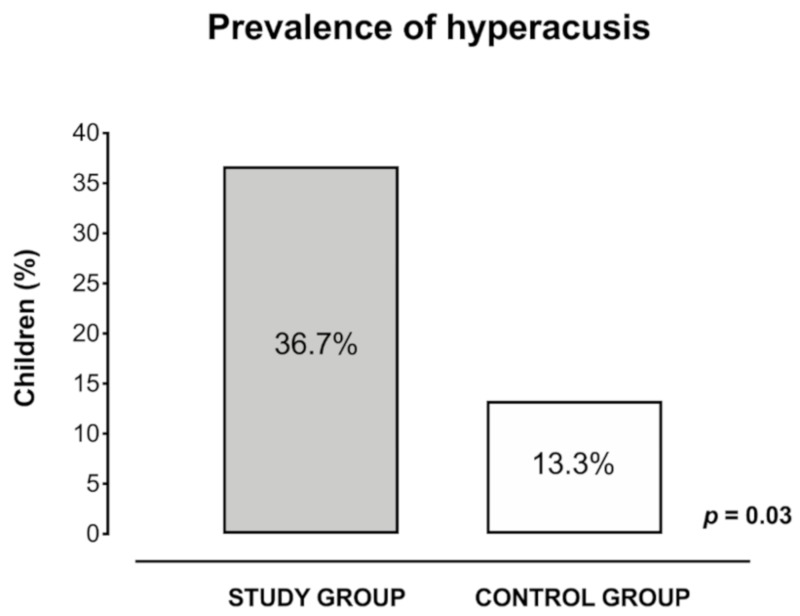
Prevalence of hyperacusis in children in the study group and control group calculated with positivity to both parent’s questionnaire and children’s interview. Prevalence of hyperacusis was 36.7% (*n* = 11) in the study group and 13.3% (*n* = 4) in the control group. Difference was statistically significant (*p* = 0.03).

Prevalence of hyperacusis was 36.7% (*n* = 11) in the study group and 13.3% (*n* = 4) in the control group. Difference was statistically significant (*t* = 2.13, *p* = 0.03, *d* = 0.55) (Figure 5).

## 4. Discussion

The present study shows that the prevalence of hyperacusis in the study and control groups was, respectively, 36.7% and 13.3%. These preliminary results, even if obtained from a small sample, suggest that hyperacusis may have a higher prevalence in children with a diagnosis of ADHD compared to otherwise healthy children. The majority of children included in the study were males; this is consistent with previous studies that reported an increased prevalence of hyperacusis in boys compared to girls [12,16].

Comparing the prevalence of the control group with the data present in the literature, our prevalence (13.3%) is within the range of 3.2–17.1% identified in the Rosing review [31].

The higher prevalence of hyperacusis found in the study group suggests a potential association between ADHD and hyperacusis as already hypothesized by other authors [12,15,33], and is in line with the evidence that hyperacusis can be more frequent in children affected by neurodevelopmental disorders, in particular ASD [12,33,35,36,39,40,41,42]. Indeed, although different nosological entities, it is important to consider the relationship between ASD and ADHD: up to half of the children affected by ASD also present with ADHD (20–85%) and 30–65% of children with ADHD show autistic traits [43].

The most common responses to sounds in both groups were, in order of frequency: covering the ears for the duration of the sound, running away from the sound, and complaining of pain in the ears. In the study group, these reactions were more common compared to the control group. In our cohort of children, the most common annoying sounds included classroom noise, screams, school bells and bombs, in accordance with the sounds capable of causing hyperacusis observed in other studies [13,33]. Another observation is that the most annoying sounds were those that came from uncontrollable sound sources or were unpredictable.

The mechanisms underlying hyperacusis in children are unknown in most cases and are even more difficult to identify in patients with neurodevelopment disorders [5,44,45,46,47]. In children affected by ASD, Khalfa hypothesized that hyperacusis can be the consequence of disordered loudness processing with a restricted dynamic range of perception, an increased subjective perception and reduced tolerance of loudness. This auditory hypersensitivity is found not only with loud sounds but also with sounds considered to be of moderate sound intensity [40]. Another important pathological mechanism causing hyperacusis and tinnitus in children with neurodevelopment disorders could be the sensory processing [36,48,49]. These patients may have difficulties in regulating or integrating sensory information (visual, touch, sounds, smells, proprioception), which can lead to patterns of hypersensitivity to sensory stimuli or a sensory overload effect [50,51,52,53,54]. This may be due to Sensory Over-Responsivity (SOR), a sensory modulation disorder manifested by behavioral responses that are faster, longer or more intense compared to peers [55]. SOR has been correlated in several studies with neurodevelopmental disorders such as ADHD and ASD [56,57,58,59,60]. Moller and Rollins [61] have hypothesized that in these children, hyperacusis may be the result of an alteration of non-classical auditory pathways with involvement of the limbic system. This could explain the intense emotional reactions in response to exposure to the troublesome sounds and could be related to an irritability condition that could have worsened children’s reactions to sound. 

Another important element is the relationship between hyperacusis and attention and cognitive functioning in children with ADHD. Children with a diagnosis of ADHD have difficulty in maintaining attention on specific tasks and inhibiting visual and sound distractors [62,63,64]. Such inability to inhibit an answer may explain many of the behavioral symptoms, including hyperactivity, impulsiveness, hyperacusis, and cognitive symptoms [62,63,64]. Fabio et al. analyzed the characteristics of auditory vigilance in ADHD subjects with and without interference, evaluating if deficits of the executive function are at least partly due to a deficit in automatic processing, and reported that children with ADHD exhibit a deficit both in automatic and controlled processes [65,66].

The diagnosis of hyperacusis in a pediatric age is complex and not yet standardized, and consists of three phases: medical history, audiological evaluation and use of specific questionnaires [12,14,33]. Other proposed evaluation methods include the study of loudness discomfort levels and of the stapedial reflex [13,67]. In the authors’ opinion, the investigation of possible hyperacusis in children should be mainly based on anamnestic history, observation and questionnaires aimed at collecting information about reactions to troublesome sounds and situations, use of safety behaviors, and impact of sound exposure on the activities of the child and the family. The lack of a universal agreement on the usefulness of the loudness discomfort levels and the use of the stapedial reflex to investigate hyperacusis led us to the choice not to use them in the audiological diagnostic phase, as they are more likely to lead to distress than give significant information [33].

Evidence on the treatment of hyperacusis in children is still fragmented. Amir et al., reported good therapeutic results with behavioral therapy, in which reassurance and explanation of the condition tailored to the child’s individual characteristics was used to assist children in developing their own coping mechanisms and relaxation strategies; in most cases, this approach was also combined with sound therapy [12].

### Limits of Our Study

Our study presents several limits. The main limit is the small size of our sample; larger studies are necessary to confirm our results. Secondly, the study design does not allow differentiation between hyperacusis, misophonia and phonophobia, leading to a possible confusion between these conditions. Similarly, SOR has not being investigated in the present study. Only children with a diagnosis of ADHD, combined presentation, were included to make the sample more homogeneous, while children with predominantly inattentive or predominantly hyperactive/impulsive ADHD presentations were excluded. Hyperacusis has been evaluated using a questionnaire; this tool has been modified and translated into Italian from the one validated by Coelho in 2007 [13]. No objective methods such as LDL and acoustic reflex studies have been used to assess hyperacusis because their use is still debated in children with hyperacusis. Lastly, the questionnaire has not been validated yet in the Italian language; future studies will contribute to validation.

## 5. Conclusions

The preliminary results of our study confirm a higher number of children with hyperacusis among those with ADHD compared to that of the general population of a similar age. The higher prevalence of hyperacusis in ADHD suggests that performing a neuropsychiatric assessment in all children complaining of hyperacusis who come to the attention of the otolaryngologist/audiologist would be useful. Similarly, it is advisable that all children with ADHD undergo an audiological evaluation to investigate the presence of hyperacusis. In fact, hyperacusis may worsen the typical symptoms of ADHD, resulting in a deterioration of the quality of life for children and their families and may contribute to the failure of cognitive-behavioral therapy. More studies on larger samples are necessary to confirm our preliminary data.

## Figures and Tables

**Table 1:** Parent’s questionnaire.

Answer the Following Questions	
1.	Do you think that your child is too sensitive to every day’s sounds?
2.	Is there any sound that your child dislikes?
3.	Is there any sound that your child considers painful?
4.	Is there any sound that scares your child?
Indicate Your Child’s Most Frequent Reaction to Loud Sounds	
5.	Cover ears
6.	Cries
7.	Escapes from sound
8.	Steps back to avoid sound
9.	Says “I don’t like it” or “It hurts”
10.	Other

**Table 2:** Children’s interview.

Answer the Following Questions				
1.	Can you hear well?			
2.	Do you hear a noise inside your ears or head?			
3.	Are you bothered by any kind of sound or noise?			
Do any of the Following Sounds Annoy you?				
School recess	TV	Car	Toys	Firecrackers
Classroom noise	Radio	Motorcycle	Balloons	Bombs
Screams	Mixer	Truck	Whistle	Thunder
School bell	Telephone	Ambulance	Musical instruments	Dogs

**Table 3:** Answers from children in the study and control group.

Are You Annoyed by “…”	Number of Children that Considered the Sound Annoying	
	Study Group (*n* = 30)	Control Group (*n* = 30)
School recess	10 (33.3%)	4 (13.3%)
Classroom noise	16 (53.3%)	6 (20%)
Screams	16 (53.3%)	8 (26.7%)
School bell	11 (36.7%)	6 (20%)
TV	3 (10%)	3 (10%)
Radio	3 (10%)	1 (3.3%)
Mixer	8 (26.7%)	4 (13.3%)
Telephone	2 (6.7%)	1 (3.3%)
Car	5 (16.7%)	3 (10%)
Motorcycle	6 (20%)	4 (13.3%)
Truck	5 (16–7%)	3 (10%)
Ambulance	4 (13.3%)	2 (6.7%)
Toys	6 (20%)	3 (10%)
Balloons	10 (33.3%)	5 (16.7%)
Whistle	8 (26.7%)	4 (13.3%)
Musical instruments	8 (26.7%)	4 (13.3%)
Bombs	13 (43.3%)	7 (23.3%)
Firecrackers	9 (30%)	6 (20%)
Thunders	10 (33.3%)	8 (26.7%)
Dogs	8 (26.7%)	4 (13.3%)

**Table 4:** Identification of children with hyperacusis based on parent’s questionnaire and children’s interview.

ID #	Study Group			ID#	Control Group		
	Parent’s Questionnaire	Children’s Interview	Bothered by Sounds (Y/N)		Parent’s Questionnaire	Children’s Interview	Bothered by Sounds (Y/N)
1 *	14	11	Y	31	4	2	N
2 *	16	7	Y	32	2	3	N
3	0	1	Y	33	0	3	N
4	0	0	N	34	4	3	N
5	4	10	Y	35	2	3	N
6 *	8	5	Y	36	4	2	Y
7 *	12	7	Y	37	4	4	N
8	0	7	Y	38 *	8	6	Y
9	2	0	Y	39	0	1	N
10 *	16	11	Y	40	2	5	N
11 *	9	5	Y	41	6	0	N
12	12	3	Y	42	6	1	N
13	0	2	N	43	2	2	N
14 *	12	8	Y	44	0	0	N
15	0	5	Y	45	4	2	N
16	4	7	Y	46	2	0	N
17	0	1	N	47	2	2	N
18	8	4	Y	48 *	10	5	Y
19	0	2	N	49	0	1	N
20	4	5	Y	50	2	0	N
21 *	12	11	Y	51	2	4	N
22	4	3	Y	52	6	1	N
23 *	14	7	Y	53	4	3	Y
24	6	0	N	54	4	0	N
25	0	4	Y	55 *	10	8	Y
26 *	9	6	Y	56	2	6	Y
27	4	5	Y	57	2	3	N
28 *	12	11	Y	58	2	6	N
29	4	3	Y	59 *	8	6	Y
30	8	4	Y	60	0	5	N

Hyperacusic children are marked with asterisk (*).
